# In the name of the rose: a roadmap for rose research in the genome era

**DOI:** 10.1038/s41438-019-0156-0

**Published:** 2019-05-03

**Authors:** Marinus J. M. Smulders, Paul Arens, Peter M. Bourke, Thomas Debener, Marcus Linde, Jan De Riek, Leen Leus, Tom Ruttink, Sylvie Baudino, Laurence Hibrant Saint-Oyant, Jeremy Clotault, Fabrice Foucher

**Affiliations:** 1 Plant Breeding, Wageningen University and Research, P.O. Box 386, 6700 AJ Wageningen, The Netherlands; 2 Faculty of Natural Sciences, Institute for Plant Genetics, Molecular Plant Breeding, Leibniz University of Hannover, Herrenhäuser Strasse 2, 30419 Hannover, Germany; 3 ILVO, Plant Sciences Unit, Flanders Research Institute for Agriculture, Fisheries and Food, Caritasstraat 39, 9090 Melle, Belgium; 4 BVpam CNRS, FRE 3727, UJM-Saint-Étienne, Univ. Lyon, Saint-Etienne, France; 5 IRHS, Agrocampus-Ouest, INRA, Université d’Angers, SFR 4207 QuaSaV, 42 rue Georges Morel BP 60057, 49 071 Beaucouzé, France

## Abstract

The recent completion of the rose genome sequence is not the end of a process, but rather a starting point that opens up a whole set of new and exciting activities. Next to a high-quality genome sequence other genomic tools have also become available for rose, including transcriptomics data, a high-density single-nucleotide polymorphism array and software to perform linkage and quantitative trait locus mapping in polyploids. Rose cultivars are highly heterogeneous and diverse. This vast diversity in cultivated roses can be explained through the genetic potential of the genus, introgressions from wild species into commercial tetraploid germplasm and the inimitable efforts of historical breeders. We can now investigate how this diversity can best be exploited and refined in future breeding work, given the rich molecular toolbox now available to the rose breeding community. This paper presents possible lines of research now that rose has entered the genomics era, and attempts to partially answer the question that arises after the completion of any draft genome sequence: ‘Now that we have “the” genome, what’s next?’. Having access to a genome sequence will allow both (fundamental) scientific and (applied) breeding-orientated questions to be addressed. We outline possible approaches for a number of these questions.

## Introduction

Rose is the most well-known and beloved ornamental plant worldwide. As in most other ornamental plant breeding programmes, molecular tools have up to now rarely been used. There is a number of obstacles to implementing molecular breeding in roses. These include its tetraploid nature, the fact that it is vegetatively propagated and that large genetic gains can still be achieved by simple crossing and selection. Moreover, a significant gap exists between research and breeding practices, impeding the application of developed genetic tools in practice. While for large agricultural crops the primary focus is on yield and other quantitative traits for both the academic community and breeders, rose research has focussed on both characteristics that are qualitative, such as presence/absence of the ‘double flower’, and some disease resistances, and on complex qualitative traits, including flower colour, scent emission, bud outgrowth^1^, floral development and vernalization response^2^. Although rose is an ideal model species for studying the molecular basis of these traits, they are easily examined by eye by breeders. For instance, scent is a complex trait that is being studied, but it is examined by an experienced nose in a breeding programme.

The worldwide rose research community is relatively small. Therefore, the development of new information and tools for rose breeding and genetics in the past few years has been an important step. The availability of rose genome sequences^3–5^ has made the identification of candidate genes for these traits possible. Once a candidate gene has been found, allelic diversity may be linked to functional diversity (see ref.^6^), leading to ‘candidate alleles’, which is of much use in a highly heterozygous and polyploid crop. Next-generation sequencing has also facilitated the creation of transcriptomes and large numbers of single-nucleotide polymorphism (SNP) markers, which can be assessed using the 68k WagRhSNP array for rose^7^ or by single SNP assays using flanking sequence information. Owing to newly developed software for dosage scoring and genetic mapping in polyploids, ultra-dense genetic maps have recently been produced in diploid^5^ and tetraploid rose^8,9^ that contain many more markers than maps from previous diploid rose populations (collated in ref.^2^). These maps have enabled subsequent quantitative trait locus (QTL) mapping studies^10^ and genome-wide association analyses^11,12^ in tetraploid rose, helping to identify regions of the genome that are statistically correlated with traits of interest. As the genomes and most of the resources are publicly available, rose genomics is far more accessible than ever before.

Here we discuss the opportunities for better understanding of structural variation in the rose genome and genome evolution in the genus *Rosa* and applications for QTL mapping, genome-wide association study (GWAS) analysis and functional analyses of traits, and for measuring genetic diversity, which, we hope, will ultimately improve the speed and precision of breeding new rose cultivars.

## Genome sequence and genomic tools in rose

### The rose genome sequences

Nakamura et al.^3^ released the first rose genome sequence from the wild and heterozygous *Rosa multiflora*. It was still a genome in pieces (low N50 and 83,189 scaffolds, Table 1). The recent release of two high-quality reference genomes, obtained by sequencing haploids using a combination of short and long reads, represents a tremendous improvement with N50 of 3.4 and 24 Mb and 551 and 82 scaffolds, respectively (Table 1). Pseudomolecules, corresponding to the chromosomes, were obtained by anchoring the new sequences to a high-density diploid SNP genetic map^5^ or to the high-density map for tetraploid rose of Bourke et al.^8^, and validated by HiC sequencing^4^. Details concerning genes and transposable element annotation are listed in Table 1. The genomes are publicly available at GDR^13^, NCBI and some dedicated websites (Table 1).

**Table 1:** Principal metrics of the recent published rose genome sequences

Genotype	Haploid of ‘Old Blush'	Haploid of ‘Old Blush'	*Rosa multiflora* Thunb.
Methods	Illumina/PacBio	Illumina/PacBio	Illumina
N50	3.4 Mb	24 Mb	90.8 kb
No. of contigs	551	82	83189
Total genome size	512 Mb	515 Mb	740 Mb
Annotated genes			
Coding	39,669	36,377	67,380
Non-coding	4812	3971	ND
Transposable elements	61.8	65.2	56.4
Class I	35.1	31.6	12.3
Class II	11.7	11.6	2.4
Other	15	22	39.2
Reference	^5^	^4^	^3^
Dedicated websites	https://iris.angers.inra.fr/obh/	https://lipm-browsers.toulouse.inra.fr/pub/RchiOBHm-V2/	http://rosa.kazusa.or.jp/

N50 represents the length of the contig in order to have 50% of the total assembly contained in contigs larger than this value. ND for non determined

**Fig. 1:**
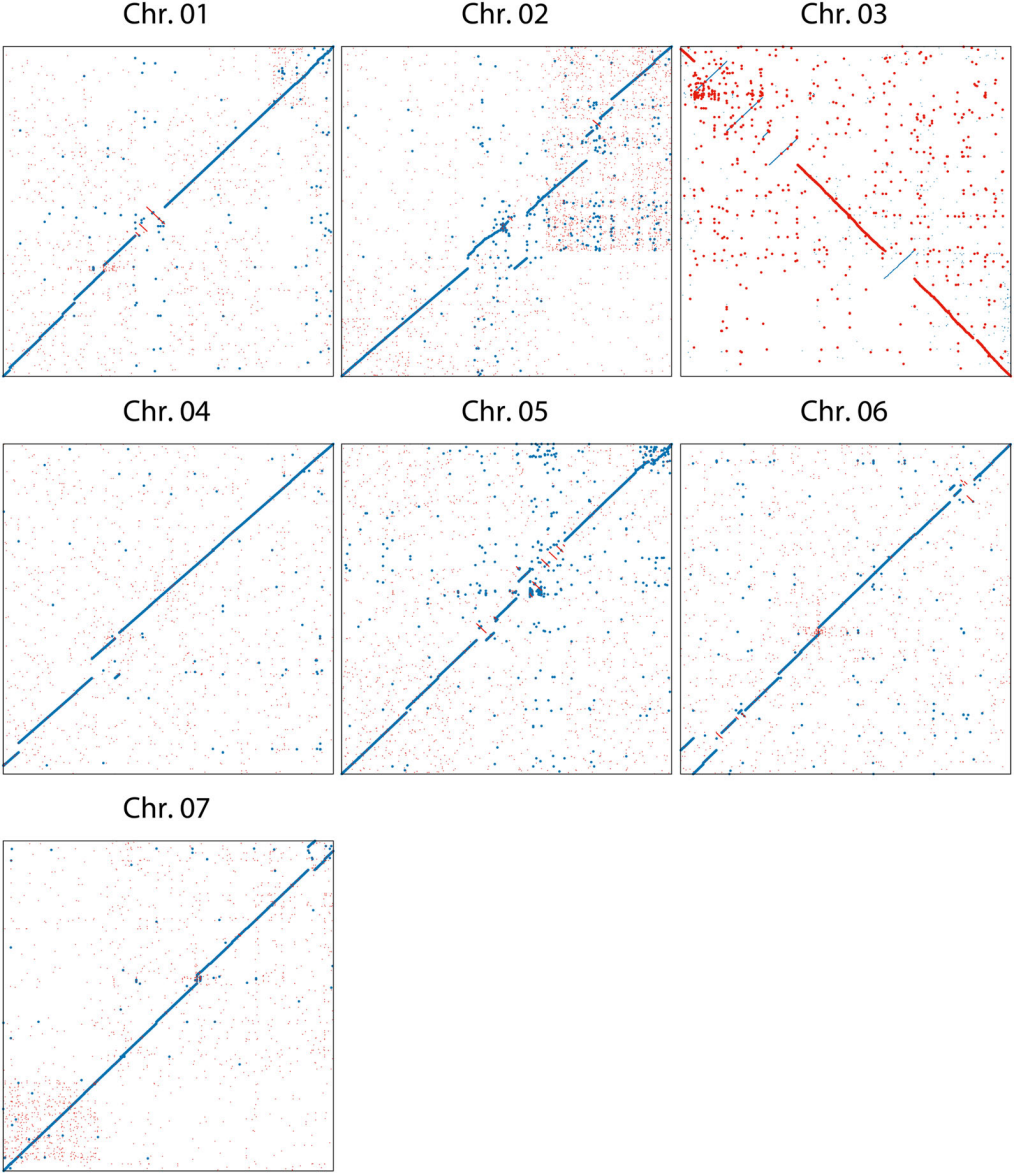
Comparison between the two *Rosa chinensis* ‘Old Blush’ sequences released in 2018. On the *x*-axis, the OBDH_1.0 Angers assembly (accessed from https://iris.angers.inra.fr/obh/) versus, on the *y*-axis, the RchiOBHm-V2 Lyon/Toulouse assembly (accessed from https://lipm-browsers.toulouse.inra.fr/pub/RchiOBHm-V2/). Blue points represent forward alignments, while red dots represent reverse alignments, with a minimum length of 250 bp. The alignment was performed using MUMmer v3.23^159^. Chromosome 3 of the RchiOBHm-V2 assembly is in the orientation of the ICM map of Spiller et al.^2^. The rearrangement at two-thirds of chromosome 3 is around the *RoKSN* locus^5^

A comparison of the two independent assemblies of the haploids of *Rosa chinensis* ‘Old Blush’ indicates that at they are essentially co-linear, indicating a high level of accuracy at both the contig and scaffolding level. Some assembly discrepancies appear as rearrangements (Fig. 1). These rearrangements may be due to assembly problems or they may highlight structural differences between the two haplotypes in ‘Old Blush’, which has been described as an interspecific hybrid^14^. One local rearrangement that has been studied in detail is at the continuous-flowering locus, where a large inversion led to the loss of the *RoKSN* gene (Fig. 1, ref.^5^). The *R. multiflora* genome^3^ has not been compared to the other two as it is much more fragmented.

### Transcriptomics resources

Next-generation sequencing has also facilitated the creation of transcriptome data of various tissues and developmental stages for several genotypes and species (e.g. see refs.^15–18^). Such data can be further used to perform gene curation and annotation. As more and more transcriptomic data will be available with their associated metadata, a gene expression atlas can be developed; such tools will help to select for candidate genes and perform meta-analysis.

### Molecular markers

SNPs are abundantly present as genetic variation between and within accessions, their detection can be automated and they are easily linked to genome sequences (based on the flanking regions). Therefore, they are currently the marker of choice. Large numbers of SNP markers have been generated in rose using genomic or transcriptomic sequences^19,20^. The 68k WagRhSNP Axiom SNP array^7^ was designed to genotype more than 68,000 SNPs identified from transcriptome sequences from 12 modern garden roses, two cut rose cultivars and a *R. multiflora* hybrid^7^. PCR-based marker systems, for example, KASP (Kompetitiv Allele-Specific PCR) can be used for single SNP marker genotyping as well. Since many SNP markers are polymorphic in both garden and cut roses, and in diploid as well as tetraploid roses, there are now many publicly available resources to identify regions in the genome that are associated with traits of interest.

Current and future comparisons across studies will benefit if this SNP array, or a subset of the SNPs on the array, is used for genotyping across different studies. For instance, it would become possible to expand the sample size of a GWAS study, or to do a combined re-analysis on data from independent GWAS panels.

Biallelic SNPs can provide similar levels of discerning power as multi-allelic simple sequence repeat (SSR) markers if a sufficiently large number of SNP markers is employed. The power to distinguish multiple alleles at a locus may also be increased by combining a number of neighbouring SNP markers into a single multi-allelic marker and analysing them as haplotypes^21^. Haplotypes also carry information on identity by descent. The first strategies have been developed to infer phasing of haplotypes from genotype data; however, they have a low efficiency when ploidy increases^22^. Sequencing technologies such as Pacific Biosciences and Oxford Nanopore produce longer sequencing reads, and therefore also longer (and potentially more informative) haplotypes.

Re-sequencing for diversity analysis avoids possible ascertainment bias while offering allele dosage estimates from read proportions^23–25^, although various steps in the actual protocol used may negatively affect the reliability of dosage estimation in polyploids. Re-sequencing at very low coverage per individual (skim sequencing) or the use of a complexity reduction step^26^, such as restriction-site-associated DNA sequencing or genotyping by sequencing (GBS), has been proposed to reduce cost, and indeed have already been applied in rose^5,27,28^. Population genetics analysis that do not require individual genotype information (Pool-seq) reduces the number of libraries, while protocols for reducing the costs of libraries have also been developed^29^.

As most cultivated roses are complex hybrids, Insertion/Deletion variants (InDels) occur frequently^5^. Indels thus represent an interesting source of genetic variation. Re-sequencing will include them; however, indels are difficult to detect in a reliable way as variant calling programmes often disagree on the detection of indels. Hence, the quality (reproducibility) is often much lower than for SNP calls. In Citrus it was shown that indels can help account for the contribution of ancestral species^30^.

### Genotyping and genetic mapping

SSR (or ‘microsatellite’) markers have been widely employed in genetic diversity and mapping studies in rose. Several genetic maps have been produced with SSR markers and other markers, including amplified fragment length polymorphism (AFLP) and nucleotide binding site (NBS) to map traits, mostly in diploid crosses. These maps have been integrated by Spiller et al.^2^ into a consensus map. The numbering and orientation of this map, with annotated traits, have subsequently been used for high-density SNP array-based maps, which in turn were used for anchoring and ordering the rose genome sequences, thus tying together most genetic mapping and QTL studies in rose. As the SNP markers were derived from expressed sequences, this gave a focus on the part of the genome where most genes reside and where most recombinations occur during meiosis, and thus to a high-quality haploid genome assembly in those regions that are of the highest interest.

For polyploids, SNP array data currently provided the clearest information on the allele dose of markers. Using the dosage of SNP markers, tetraploid SNP genotypes can be used for linkage analysis with dedicated polyploid mapping software such as TetraploidSNPMap^31^ or polyMapR^10,32^. Ultra-dense genetic maps thus have recently been produced in diploid^5^ and tetraploid rose^8,9,33^, either for separate homologous chromosomes, or integrated across chromosomal linkage groups (LGs). Yan et al.^27^ made a map using SNPs in GBS data. The dense SNP maps have enabled subsequent QTL mapping studies^34^ and genome-wide association analyses^11,12^ in tetraploid rose, helping to identify regions of the genome that are statistically correlated with traits of interest.

## Genetic diversity in rose

Historical rose breeding involved a complex sequence of interspecific hybridizations between seven to fifteen wild species that contributed to the germplasm of modern roses (the number depends on the interpretation and importance attributed to their contribution to the domestication), thus shaping the genomes of modern cultivars after three centuries of breeding^35,36^. This atypical genetic bottleneck means that genetic diversity in roses now encompasses two sources of sequence variation. On the one hand, genetic diversity in the genus *Rosa* comprises 140 to 180 wild species, including all genetic variation therein; on the other hand, it comprises a tremendous number of rose cultivars, currently estimated to exceed 30,000 cultivated cultivars^37^.

Several studies on genetic diversity in rose have been performed with SSR markers^38–42^, mostly in the cultivated germplasm. Phylogenetic studies have been conducted on *Rosa* species across the genus, and on modern rose cultivars, indicating close relationships between different botanical sections and horticultural groups (refs.^42–48^). These studies have shed some light on general patterns of genetic diversity, but give little information on the contribution of specific parents in hybridizations to desired traits.

### Rose domestication

Two important rose species can be considered to be domesticated in the common sense of the term: the tetraploid *Rosa gallica* in Europe^49^ and the diploid *R. chinensis* in Asia^39^. The most remarkable recent insights in the origin of modern cultivated roses concern the study of the genetic structure of old Chinese garden roses^39^, their large introgression in the European germplasm since the eighteenth century^42^ and the subsequent selection during the twentieth century within a subsample of these hybrid garden roses to obtain cut roses^40^. In contrast, almost nothing is known on the origin of the roses that are used for the production of essential oils: *Rosa damascena* and *Rosa centifolia*.

A comparative analysis of these two domestication events in different parts of the world has not been carried out yet. It would involve wild and early cultivated genotypes of the two species and may reveal the precise location of domestication events by comparison to wild gene pools, the presence of potential genetic bottleneck(s) during the domestication event and even the timing of domestication events. Generally, the domestication of perennial species, often with long intergenerational time, is more recent than most annual crops and involves shorter or even no genetic bottlenecks, leading to a reduced domestication syndrome. Rose, as a perennial species with a short juvenile phase, may have an intermediate pattern. Next to testing the impact of ploidy level on domestication dynamics, the comparison of regions targeted by selection in *R. gallica* and *R. chinensis* and the domestication syndrome traits in common between the two species (double flower, colour diversification) or specific to one of the species (recurrent blooming in *Rosa chinensis*, abiotic stress tolerance in *Rosa gallica*) could clarify the phenomenon of convergent or differential domestications. As in other perennials, including apple, citrus and olive^50–52^, introgressions may have played an important role in the domestication of rose.

### Mosaic genomes in modern roses

Species hybridizations may have led to ‘mosaic’ genomes, which are formed by small parts of discontinuous ancestral genomes, as has been observed in cultivated *Citrus*^50^. Re-sequencing of the first modern rose cultivar ‘La France’ and three or four species each from the sections *Cinnamomeae*, *Synstylae* and *Chinenses* showed that large regions of chromosome 2, 3 and 5 of the triploid cultivar ‘La France’ have a strictly *Chinenses* origin, while other regions of chromosome 2, 5 and 7 had a *Synstylae* origin only^4^. This study on ‘sequence signatures’ is the first step towards a more comprehensive understanding of the history of the rose breeding process.

Owing to the vegetative propagation of cultivars since their commercial introduction, the original genome constitution can still be accessed and sequenced and thus 300 years of rose breeding history can be reconstructed. Sequencing of founder individuals of subsequently developed horticultural groups, or cultivars with great commercial or breeding success, may reveal the origin of ancestral genome segments (sequence signatures). Reconstructing the segregation of these ‘founder haplotypes’ throughout centuries of breeding as captured in cultivar collections, while correlating these genomic segments with the evolution of horticultural traits in roses, may identify signatures of selection. In addition to ‘La France’, a broader study of various Tea hybrids may show if the observed patterns of single ancestry of specific chromosomic regions were targeted by breeders or if they appeared by chance, by researching over-representation of a given ancestry in the pedigree. This approach has already been used in apple, peach, cherry and strawberry to identify haplotypes passed on by highly successful parents during the breeding process^53–59^. Identification of genealogical relationships between cultivars^60^ can be used for validation.

Understanding the mosaic genomes of modern cultivated roses may also detect the occurrence and determine the impact of structural divergence between hom(e)ologous chromosomes during meiosis. A study of pairing behaviour between tetraploid rose parents of a segregating population showed evidence for ‘segmental allotetraploidy’: pairing of some chromosomes was preferential (partially disomic) while most other chromosomes behaved tetrasomically^8^. Sometimes, these two modes of inheritance were observed in segments of the same chromosome, in the meiosis of one of the parents. A comparison of chromosome pairing behaviour during rose meiosis and the occurrence of structural variation between hom(e)ologous chromosomes across several meiosis in different genetic backgrounds is needed in order to understand the link between these two phenomena, including which type(s) of structural variation exist. Alternatively, the behaviour may be genetically governed. In the latter case the mechanism may not be rose-specific. Nevertheless, rose is a suitable model crop for such studies as several species and much historical material (old cultivars produced involving species crosses) is available. A broad analysis of the modes of inheritance in various modern rose cultivars, but also in old garden roses and wild roses, should clarify whether ‘segmental allotetraploidy’ occurs commonly in the genus *Rosa* or whether it is genotype-specific, and whether it occurs more frequently in some subsets of the rose germplasm than others.

### Sports

Rose cultivars are clonally multiplied through vegetative propagation. However, mutations occur frequently, leading to variation, sometimes visible phenotypically (‘sports’). Most of this variation is thought to be due to point mutations, although transposon activity and epigenetic effects cannot be ruled out. Consistent with point mutations, original cut rose varieties and mutants thereof were identical with 11 SSR markers, so mutant families could easily be detected, while all seedling-derived varieties could be clearly distinguished from them and from each other (similarity <0.90)^61^.

Comparing the genome of pairs of sport and original cultivar may provide information on genomic regions that explain mutant phenotypes and help to understand why some traits are more often involved in sports, such as climbing growth habit or petal colour changes.

### The Canina meiosis

Species of the *Caninae* section display a unique type of asymmetric meiosis that has not been described in other angiosperm species^62,63^. It also appears in *Alba* roses, a European Old garden rose group, which could be hybrids between sections *Caninae* and *Gallicanae*^64^. The *Caninae* section is composed of tetraploid, pentaploid and hexaploid species. Irrespective of the ploidy level, the *Caninae* asymmetric meiosis involves the formation of one set of bivalent-pairing chromosomes and the remaining chromosomes are univalent (unpaired). One reduced set of bivalent chromosomes and all the univalent chromosomes are included in an egg cell, while the other reduced bivalent chromosome set is transmitted by pollen cells. It was shown by molecular markers and fluorescent in situ hybridization that the two bivalent-forming chromosomes are highly homologous, whereas univalent chromosomes are more divergent^65,66^. Genome sequencing of several species of the section *Caninae* may help to reveal whether structural variation among bivalent and univalent chromosomes is the primary cause of asymmetric meiosis or if and how the decision per chromosome to pair or not to pair is genetically controlled. Inheritance of haplotypes or complete chromosomes through pollen and egg cells can be most efficiently studied using a dense array of SNP markers. The software package polymapR can deal with odd ploidies and with offspring of plants with different ploidy level^10,32^. Based on the offspring data preferential chromosomal pairing in each parent can be detected, and all meiotic recombination events would become visible.

## Structural genomic variation in rose

### Interspecific genomic variation

In the genus *Rosa* the 2C DNA amount in diploid species ranges from 0.78 pg in *Rosa xanthina* (section *Pimpinellifoliae*) to 1.29 pg in ‘Félicité and Perpétue’ (hybrid Sempervirens)^67^ and from 0.73 pg in *R. zhongdianensis* (section *Pimpinellifoliae*) to 1.77 pg in *R. brunonii* (section *Synstylae*)^68^. The recently sequenced *R. chinensis* is among the largest genomes known among diploid roses (estimated at 1.16 pg^67^ to 1.67 pg^68^). Roses from the *Pimpinellifoliae* section generally have the smallest genome size, while *Synstylae* roses have the largest genomes^68^.

Genome size variation in angiosperms is typically associated with two types of events: whole-genome duplication (WGD) or transposable element amplification^69,70^. With regard to the latter, approximately 68% of the *R. chinensis* reference genome sequence consists of transposable elements, especially long-terminal repeat retrotransposons like *Gypsy* and *Copia* elements^5^. For most transposable element families, a two-fold higher abundance was found in *Rosa* as compared to *Fragaria vesca*^5^, explaining a substantial part of the genome size difference between *R. chinensis* and *F. vesca*. Shallow shotgun sequencing of a comprehensive sets of species across the genus *Rosa* and subsequent clustering and quantification of repetitive sequences will reveal if species-specific repetitive elements exist. More extensive (re)sequencing will be needed to ascertain whether differential amplification of transposable elements can explain the variation in genome size among rose species.

Besides the role of transposable elements in genome size evolution, the insertion of *copia* elements into specific protein-coding genes^5,71^ gave rise to two of the most important horticultural traits: double flower and recurrent blooming (see below). Rose breeding in general may have favoured species from sections with large genomes, as transposon activity is known to create allelic diversity^72^. A related question is whether retrotransposon activity is higher in tetraploid taxa compared to diploids, and whether this has also led to functional variation that is useful for the ornamental value of roses.

Whole-genome assembly and gene prediction has revealed no signs of recent WGD events in *R. chinensis*^4,5^. In addition, despite the difference in genome size, the 240 Mb *F. vesca* genome contains 34,809 predicted genes^73^, while the 560 Mb ‘Old Blush’ *R. chinensis* genome has only fractionally more predicted genes (39,669 genes^5^; 36,377 genes^4^).

### Rose comparative genomics

From an evolutionary point of view, rose is a very interesting model species as it includes species at several ploidy levels as well as many cultivars with a hybrid origin^74^. The sequencing of thousands of individuals in *Arabidospis thaliana*^75^, rice^76^ and maize^77^ has demonstrated extensive differences in genome constitution even among accessions within a species, leading to the definition of ‘core’ genes (which are present in all members of a species), and ‘distributed’ or ‘dispensable’ genes (present in a subset of members). The ‘pan-genome’ represents the full genome complement across all sampled members.

Metagenome-like assembly strategies in rice^76^ and an analysis of 3010 re-sequenced rice accessions^78^ revealed that ‘distributed’ gene families showed enrichment in regulation of immune and defence responses. Other studies also unveiled their role in adaptation to abiotic and biotic stresses^79^, species diversification and development of novel gene functions^80^. (Meta)genome assembly and gene annotation of species across the *Rosa* genus and in closely related species, and subsequent comparative genomics will be required to define whether rose also has conserved and lineage-, section- and/or species-specific genes. One of the possible hypotheses is that resistance (*R*) genes behave as a dispensable group gene family, while susceptibility (*S*) genes generally would be members of core gene families. For such questions to be answered, it will be necessary to collect and analyse genome sequences of species and accessions across the genus that represent the taxonomic diversity, but also the diversity in various abiotic and biotic conditions in which roses grow.

Among the most interesting cases to study in roses is the question of resistance genes. The sequencing of bacterial artificial chromosome (BAC) clones around the *Rdr1* locus, contributing to the rose black spot resistance, in *R. multiflora* (9 TIR-NBS-LRR (*TNL*) genes) and *R. rugosa* (11 *TNL* genes) enabled both rearrangements and duplications in this locus to be discovered^81,82^. A larger analysis of clusters of resistance genes in roses is needed to understand how their organization and evolution can be associated with resistance levels.

## Important traits in rose

For decades, genetic and physiological approaches have been developed to decipher ornamental traits and to identify regions of important genes controlling these traits^83^. We will briefly summarize the most important findings and the main issues for current or future research in these traits since the publication of the rose genome sequence. We will focus amongst others on floral traits, including the mode and date of blooming (once-flowering versus continuous flowering), simple versus double flowers, petal development and flower colour. Besides an interest from developmental biology in the mechanisms, these traits are also important targets for breeders (see section ‘Rose breeding’).

### Mode of blooming: recurrent versus once-flowering

Wild roses flower in spring over a few weeks. Those that have the ability to bloom again after the first blooming are described as recurrent blooming. There are many recurrent blooming species in the ornamental sector, including Jasmine, Carnation, Hydrangea, Pelargonium and Lavender. After the first flowering period, a recurrent blooming rose has the ability to flower a second time, either continuously during the favourable season (continuous flowering habit) or later in the season, which may be occasional (occasional re-blooming habit)^71^. The continuous-flowering phenotype is caused by a mutation in a *TFL1*-like flowering repressor gene (*RoKSN*). Two alleles leading to recurrent flowering have been described: one is due to the insertion of a *copia* transposable element^71^ and the other is due to a large rearrangement at the *RoKSN* locus, leading to the deletion of the gene^5^. In both cases, the floral repressor is not produced and the roses flower continuously. In once-flowering roses, the expression of *RoKSN* is repressed in spring when the plant flowers and this is regulated by GA^84^. The RoKSN protein forms a complex with RoFD and competes with RoFT, a floral activator for repression of flowering^85^.

Presently, only extreme phenotypes have been studied (continuous versus once-flowering). Sequencing of alleles of the genes involved in flower initiation and flowering time in several cultivars and species by either whole-genome sequencing or targeted re-sequencing may improve the understanding of the molecular bases of intermediate phenotypes (equilibrium between vegetative and floral development). This may lead to the characterization of new *RoKSN* alleles or new loci controlling recurrent flowering or responding to environmental cues; for instance, by relating the genetic variation of *RoKSN* alleles to precise phenotyping of flowering (counting the number of flowers in time and space) as initiated on the *TFL1/FT* family genes by Wang et al.^86^. Association mapping across germplasm with a wide range of phenotypic differences in flowering may detect new loci. These genetic approaches can be extended by a functional analysis, such as knocking down *RoKSN* expression in once-flowering roses, as was previously demonstrated in *Fragaria*.

### Number of petals

Flowers of wild roses have five petals. During domestication and selection of roses, flowers with numerous petals, called ‘double flower’, have been selected. Up to 517 petals have been observed in a single flower^87^. It has been proposed that the double flower phenotype is due to dysregulation of the *ABCE* genes during floral development^88^. A dominant gene located on LG3^2,89^ controls single versus double petals (with the double flower phenotype being a dominant qualitative trait). Using a GWAS panel, it was proposed that this locus also controls the number of petals of the double flowers^5^, next to two QTLs on LG2 and LG5^87^. Hibrand Saint-Oyant et al.^5^ identified a rose *APETALA2/TOE* homologue within the QTL region on LG3 as most likely candidate gene for the major regulator of petal number in rose. Silencing it decreased the number of petals^90^. It was proposed that the insertion of a TE in the eight intron is responsible for transcription of a messenger RNA without a miR172 binding site, leading to a deregulation of the *APETALA2/TOE* homologue^91,92^.

The ideal number of petals for cut roses, from a commercial point of view, is between 40 and 60 petals, while a greater range occurs in commercial garden rose cultivars. Understanding the interaction between the major gene and other QTLs, in relation to environmental signals, will help to develop a model for petal number development and the effects of different combinations of alleles to obtain the desired inflorescence. For this we need to determine allelic diversity at this and other loci, look at expression differences, and experimentally test the effects of different combinations of alleles.

### Petal colour and development

Flower colour plays an important role in the attractiveness of rose flower for insects, but also for humans; colour therefore has an important aesthetic value. Most flower colours are present in the genus *Rosa*, except for blue, and a large diversity in the colour variants is available, including colour intensity and the organization of colour on the petals. These colours come from different genetic backgrounds: pink and purple from *Rosa rugosa*, and yellow from *Rosa ecae, Rosa foetida* and *Rosa hemisphaerica*. The orange colour, induced by the pelargonidin pigment, was introduced by Kordes. The darker-coloured central zone of rose flowers was probably derived from *Rosa persica*.

Yellow and orange colours are the result of carotenoid pigment accumulation, while red colours are explained by anthocyanin accumulation, mainly glycosylated xanthocyanidin^93,94^. In rose, a unique glycosyltransferase enzyme performs glycosylation at two different positions^95^. Different homologues of anthocyanin biosynthesis pathway genes are accumulated during pigment accumulation in the rose petals of ‘Old Blush’^93^. A blue/violet colour is usually the result of accumulation of delphinidin-based anthocyanins. The absence of delphinidin in rose is attributed to the lack of flavonoid 3′,5′-hydroxylase^96^. Recently, a connection between colour and scent emission was proposed through the action of SPL9-*miRNA156*, putatively controlling the synthesis of anthocyaninin and Germacren D^4^.

The genetic inheritance of flower colour was studied in different genetic backgrounds and a number of QTLs have already been identified^2,97^. Owing to a detailed annotation of carotenoid-related and flavonoid biosynthetic genes in the rose genome^3,4^, co-localization between genes and QTLs can now be investigated and candidate genes analysed. This may help to answer some intriguing questions regarding colour in rose: (i) Why is the red colour predominant in modern roses, (ii) why it is difficult to obtain a bright yellow colour and (iii) what are the origins of variegation?

### Self-incompatibility

In rose, self-incompatibility (SI)^98^ was proposed to be gametophytic as in other Rosaceae, where the S-locus is composed of genes coding for F-box and S-RNase proteins, as the male and female components of SI, respectively^99^. In rose, the S-locus was mapped on LG3^2,100^, in a region where S-RNase and F-box genes were located in the reference genome^5^. Transcripts of one S-RNase and one F-box gene accumulate in pistils and stamens, respectively. Furthermore, the OB genome sequence allowed the analysis of a region so far only covered by molecular markers linked to the SI phenotype. This region is syntenic with the S-locus in *Prunus persica*^5^.

### Fragrance

Fragrances in garden roses are very diverse and scent has always been an important trait in the selection process. However, not all marketed roses are heavily scented, despite the effort of breeders. In particular, roses bred for the cut flower market often lack scent. In modern roses or Hybrid Tea roses, scent is mainly produced by the petals, although stamens can also contribute^101^. Hundreds of volatile molecules, belonging to different biosynthetic pathways, have been isolated from rose petals. The combination of these molecules generates the particular rose scent bouquet. The biosynthetic pathways of many rose scent compounds are not completely known. The biosynthesis of 3,5-dimethoxytoluene, responsible for the ‘tea scent’ of some cultivars, involving *O*-methyltransferases, was the first to be deciphered^102,103^. The pathway leading to 2-phenylethanol (2PE) was also studied in detail, with the identification of the key enzymes phenylacetaldehyde synthase^104^ and phenylacetaldehyde reductase^105^. Recently, an alternative pathway, which is seasonally induced in summer, has been identified in roses for the production of 2PE. This new pathway uses aromatic amino acid aminotransferase^106^ and phenylpyruvate decarboxylase^107^.

Terpenoids, especially monoterpene alcohols such as geraniol, are also major constituents of rose flower volatiles, mostly responsible for the ‘typical rose scent’. Generally, terpenoid biosynthesis in plants is achieved by various terpene synthases^108^. However, with a combination of transcriptomic and genetic approaches, it was recently discovered that rose uses a terpene synthase-independent pathway. A key enzyme of this pathway is RhNUDX1, belonging to the Nudix protein family. A positive correlation was found between the expression levels of *RhNUDX1* and the production of the monoterpenoid geraniol, indicating the essential role of this protein in scent production in roses^109^. Despite these biochemical studies, knowledge on fragrance biosynthesis is still incomplete and cannot yet be used to assist breeders in scented rose selection. Indeed, very few genetic approaches, including QTL and GWAS, have been used to analyse scent genetic determinism in rose. The first published analysis of scent was performed using a segregating tetraploid rose population^110^. This analysis showed that a large proportion of offspring lacked fragrant volatile compounds, so that scent may have been lost by such a cross. Another study of genetic determinism of rose compounds was conducted in diploid roses^2^. Several QTLs influencing volatile contents were found, but no functional relationships to known candidate genes were demonstrated in this study.

Recently, Roccia et al.^6^ identified a QTL that co-localized with a gene involved in the pathway for 2PE, *RhPAAS*, the expression of which was responsible for the capacity of descendants to produce 2PE. The identification of genes underlying all QTLs and their functional characterization would be of great interest to elucidate the control of aroma volatile levels in rose petals. Next to QTL studies, association studies could be applied on scent traits. Once the key genes involved in the biosynthetic pathway of a compound are known, the major challenge is to understand their evolution in the genus Rosa and how favourable or unfavourable alleles were selected during evolution and domestication. For that purpose, more transcriptomic datasets of roses with contrasting scent profiles are needed, and re-sequencing data. Genetic analysis of diploid or tetraploid populations, in which the alleles from a strong-scented parent segregate, would enhance the power of such approaches.

### Vase life

Concerning petal development and senescence, which is an important trait for the vase life of cut flowers, Ma et al.^111^ reviewed the most important gene networks and the implication of different hormones in ethylene-sensitive flowers, among which rose features. Putative key regulating genes can now be targeted to study the effects on vase life. One way to do that may be to generate an inventory of the allelic diversity of the key regulating genes across the germplasm, followed by crossing and functional studies. As senescence may also cause susceptibility to fungal diseases such as botrytis, this type of research could also offer important links to disease resilience in garden roses.

### Disease resistance

The most important infectious diseases of roses are: downy mildew (*Peronospora sparsa*), powdery mildew (*Podosphaera pannosa*), black spot (*Diplocarpon rosae*), spot anthracnose (*Sphaceloma rosarum*), crown gall (*Agrobacterium tumefaciens*) and the rose rosette virus. Among the insect pests, rose aphids and thrips are critical for production, in particular for the production of cut and pot roses. To date, only few commercial rose cultivars have significant levels of disease resistance^112^, although it has become more important in the breeding process, especially for garden roses. The analyses of the inheritance of resistance to diseases revealed either single genes (e.g. against black spot *Rdr1–4*^33,81,113,114^, powdery mildew (mlo^115^) or QTLs (e.g. against powdery mildew^116,^^117^).

Based on the complete rose genome, resistance loci can now be linked to groups of candidate genes. This is facilitated by the fact that most *R* genes belong to the large group of *NBS-LRR* genes. Further, a complete genome enables markers to be generated from the complete set of putative *R* genes, as demonstrated by the RenSeq technique using capture-based sequencing^118^. This will speed up the localization of *R* genes^119^, also in wild rose species not yet used in breeding.

In cases where candidate *R* genes cannot be immediately identified, the genome assembly greatly facilitates the generation of additional, more closely linked SNP markers in those *R*-gene regions currently only loosely covered by molecular markers. This will be of importance for recently mapped loci, for example, *Rdr3* and *Rdr4*, where the most closely linked markers define a region of approximately 9 cM in which no further recombinations have been identified^32,^^114^.

### Plant architecture

Plant architecture relates to vigour, productivity and density (which may influence the incidence of diseases). In addition, new forms and shapes, including compact plants (for use in urban areas), may be needed to maintain the current rose market share or open up new markets. Progress in research in this area has been slow. Studies on rose plant architecture have been done using manual phenotyping of architectural components^120–122^ or with three-dimensional digitalization^123^. These analyses require fully developed plants and are time consuming. Nevertheless, they have produced a number of QTLs, and markers have been identified for these QTLs, showing the complexities of these characters. Scientific progress will depend on our ability to identify functional genes from relevant developmental pathways in these QTL regions, on whether allelic effects remain as strong when transferred into other genetic backgrounds and their interaction with environment and cultural practices. High-throughput phenotyping systems may not only make it less laborious to study plant development, but they may also pinpoint certain short stages in development in which genetic differences manifest themselves more clearly.

## Speeding up functional gene identification

Previously gene cloning was a long and laborious process. One approach involved positional cloning and looking for co-localization with candidate genes, as was done for the continuous-flowering gene of rose^71^. Alternatively, map-based cloning and BAC library sequencing was used, as was done for *Rdr1*, the first resistance gene for black spot^81,124^. The reference genome of rose^3–5^ opens new opportunities to rapidly isolate and characterize important loci by combining genetic, genomic and transcriptomic approaches. Here, we review various schemes that may accelerate the identification of functional genes and validation of the functional allele thereof in rose owing to the availability of reference genomes.

**Fig. 2:**
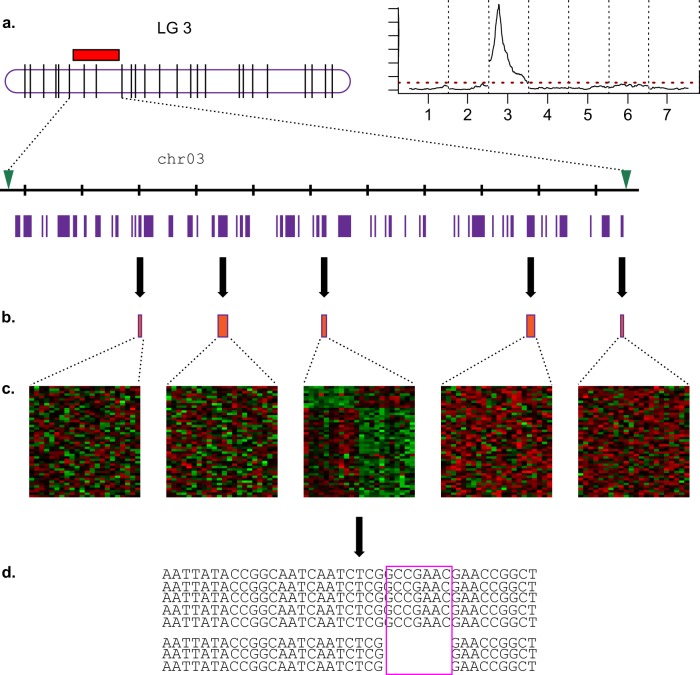
Illustrative scheme for gene cloning by combining genetic, genomic and transcriptomic approaches. **a** Using genetic approaches (such as quantitative trait loci (QTL) mapping in F1 progenies or association analysis across a panel of accessions), major genes or QTLs can be detected for important ornamental traits and located on the seven rose linkage groups. **b** In the corresponding region of the rose genome sequence, using the functional annotation of the rose genome (each purple box represent an annotated gene), putative candidate genes can be identified based on similarities with genes known to be involved in the studied process in model plants such as *Arabidospis thaliana*. In the candidate genomic region, five genes have been identified with possible roles (pink boxes), as transcription factors. **c** By transcriptomic approach, a differentially expressed gene between two contrasting conditions for the studied trait can be identified. **d** For the gene with contrasting expression, allelic variants can be identified by sequencing mutant pairs or diversity panels. In this example, two alleles are detected, which differ by an indel. Other variants can be single-nucleotide polymorphisms (SNPs) (synonymous or non-synonymous) or insertions of transposable elements

A schematic, idealized work-flow is shown in Fig. 2. A major gene, located on LG 3 of the genetic map, is identified by QTL mapping or a GWAS analysis (Fig. 2a). Flanking markers delimit the corresponding region in the genome sequence, in which five genes have been annotated that may be good candidates for the trait (Fig. 2b). Using transcriptomics, one gene (Fig. 2c) was found to be differentially expressed. By mutant sequencing (Fig. 2d) two interesting variants were identified for gene 3 (presence of an indel, which may affect the functioning of the gene). Further investigations may be necessary for validation (screening for allelic variants in a wider germplasm pool, synteny analyses or functional analyses through gene editing and/or transformation, etc.).

### Linking genetic maps with the physical sequence: demarcation of chromosomal regions

Owing to the link between genetic maps and the physical genome sequences (see section ‘Genome sequence and genomic tools in rose’), future studies may rapidly identify the sequence of flanking regions where genes of interest are located (Fig. 2a, b). Development of larger population sizes for the genetic studies will result in smaller target intervals, and hence fewer candidate genes. It is now also feasible to combine results from different genetic analyses, as demonstrated recently for the cloning of the major gene controlling the double-flower locus. For this, information from four different F1 progenies were combined to narrow down the sequence interval to a 293 kb region on chromosome 3^5^. A similar strategy could be employed for other major QTL using software such as BioMercator^125^. GWAS panels may also be combined if they share the set of SNP markers and if the phenotyping is done in the same way.

### From a sequence region to the best candidate genes

The next step is to identify the best candidate gene(s) in the previously defined genomic region, using gene annotation and knowledge of the molecular basis of the studied trait (Fig. 2b). For example, a gene known to control flower development in *Arabidopsis*, an *APETALA2* homologue, was identified in the genomic region of the DOUBLE-FLOWER locus^5^. Similarly, an F-box protein and an S-RNAse gene were identified in the region of the SI locus^5^.

Transcriptomic analyses (or meta-analysis using gene atlas) can be performed in parallel to identify differentially expressed genes, which also represent good candidates (Fig. 2c). For example, a genetic analysis identified a monoterpene synthesis locus on LG2 that co-located with a gene differentially expressed between scented and non-scented roses^109^. This led to the identification of *RhNUDX1* as a likely candidate.

Direct sequencing of mutant pairs (sports, see also section ‘Sports’) is now an option to identify the functional mutation. This could involve targeted sequencing of the gene(s) located in genomic regions known to influence the trait (with techniques such as fluidigm or multiplex amplicon sequencing), sequencing of all the coding sequences (exome sequencing^126^, targeted sequencing with capture hybridization to subsets of genes^127^) or even whole-genome re-sequencing^128^. This will potentially lead to the detection of important allelic variants, including non-synonymous mutations, insertions or deletions (Fig. 2d).

### Functional analysis

A rapid and efficient genetic transformation protocol would greatly benefit functional gene studies in rose. The first publication on rose genetic transformation was in 1994^129^. Since then, genes implied in colour, disease resistance, fragrance and architecture have been studied by stable genetic transformation in rose. In total only around 20 publications use this technique (Supplementary Table 1), probably due to the low overall efficiency (max 12%), the regeneration times required (6–12 months) and the fact that results appear to be highly cultivar-specific. Genetic information on loci and candidate genes involved in regeneration capacity were studied by Nguyen et al.^11,130^ in 96 rose genotypes, offering some hope that regeneration protocols might be streamlined in future.

To circumvent such limitations in the meantime, some rose genes have been studied in heterologous systems, including Tobacco^131^, Pelargonium^132^ and Arabidopsis^85,133–135^. Systems for transient expression in rose have been developed by agroinfiltration in petals (e.g. refs.^136,137^) or leaves^81^, and by virus-induced gene silencing in leaves, axils, seedlings^138^ and petals^139^.

Using the rose genome and inferred protein sequence data, it will be easier to identify coding sequences of genes, alternative splicing proteins, promoter and regulatory element(s). These data could be used to define CRISPR-Cas9 guide RNAs, for example, using the website CRISPOR (http://crispor.tefor.net/) on which the Rose genome sequence is available. The improvement of genetic transformation efficiency would make the use of CRISPR-Cas9 technology feasible in rose.

## Rose breeding

A strong demand for new cultivars still exists. This is reflected in the number of applications for plant breeders’ right (PBR). In the European Union (EU), the Community Plant Variety Office (CPVO) administrates PBR. Since its inception in 1991, more than 61,000 applications were filed for ornamental plants, of which 4189 were for roses (all types) (CPVO 2018: https://cpvo.europa.eu/en/applications-and-examinations/applications-and-titles-force). More roses than those listed at CPVO have been commercialized in this period, as not all roses undergo PBR application (e.g. garden roses are rarely protected by PBR). However, it is indicative of a sustained output from the rose breeding sector.

### Potential marker deployment for traits in rose

**Fig. 3:**
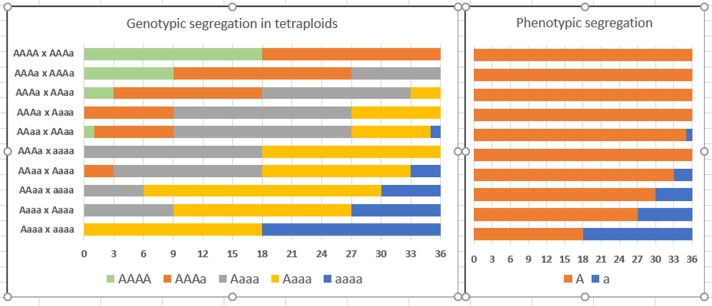
Genotypic and phenotypic segregation ratios for a trait regulated by a single dominant gene with two alleles under random bivalent pairing in a tetraploid rose. At each locus, two alleles may occur in five different allelic states (nulliplex, simplex, duplex, triplex and quadruplex). The fraction of progeny of a cross with the desired phenotype will therefore differ significantly depending of the allelic state of the parents. Crosses of one parent carrying a dominant allele in simplex configuration to a homozygous recessive (nulliplex) second genotype will result in 50% of the progeny carrying the dominant allele (in simplex configuration). In autotetraploids, dominant traits are only guaranteed to be inherited in a cross when one of the parents has at least three copies of the dominant allele. Note that additivity, multiple alleles and the occurrence of double reduction have not been taken into account.

Many of the important morphological and agronomic traits in rose are controlled by single dominant loci, for example, miniature plant habit^140^, glossy foliage^141^, resistance to powdery mildew Rpp1^115^ and black spot resistance and so on. As the phenotypes in a tetraploid progeny will strongly resemble one of the parents (Fig. 3), the dosage of the parents of the cross determines the fraction of offspring that will have the right phenotype, or be within the right range. To achieve a larger fraction of progeny carrying the trait, parents with higher allele dosages have to be combined (Fig. 3). For dominant traits allele dosage information cannot be obtained by phenotyping alone. Simplex can be distinguished from higher dosages by analysing a small progeny or an analysis of the pedigree. However, when two or more allele copies are present, the exact dosage cannot be inferred and needs be determined using linked SNP markers. Given the current strategies that breeders use for selection, the first steps in marker-assisted selection will most probably include selection of parents with optimized marker dosage for single genes of importance^142^.

Ornamental plant breeders often express concern that innovative traits may be missed by a marker-driven breeding programme. However, this assumes the breeding programme is entirely run by marker-assisted breeding without input from the ‘breeder’s eye’. This is unlikely to be the case^19^. Parental selection as outlined here only concerns optimizing the choice of the most optimal parent *pairs* out of many possible combinations of parents in view of an important trait in the offspring.

Determining the precise allelic configuration of QTLs in tetraploids is complicated, but advances in this direction have been made in recent years^34,143^. Markers whose parental phase is known (i.e. assigned to specific parental homologous chromosomes) could go a long way towards unravelling the allelic composition of important genes in the coming years. Transferring this knowledge into stable and selectable markers could be a very welcome development for the breeding community.

For quantitative traits, the marker-trait association may follow a dosage-dependent relationship if the allele dosage is additive^5^. For complex polygenic traits for which selection is not possible in the seedling stage, breeders could benefit from a better knowledge on genes that are in control, but a genomic selection approach (naive for the underlying genetics) might be useful as well. Examples of such traits are senescence (vase life), scent, disease resistance and plant architecture. One of the main criteria in cut rose selection is production, but this trait is hardly researched in roses from a genetic perspective.

### Multiple functional alleles at one locus

Outbreeding tetraploids may contain multiple alleles at any single locus. A GWAS analysis of a set of tetraploid cultivars or accessions will almost certainly include multiple alleles at one locus, but even the two parents of a controlled cross may contain more than two different alleles among the eight alleles that segregate. Separate marker assays may therefore be needed to comprehensively tag all functional alleles, which may be challenging (or even impossible) at the single SNP level. One approach to circumvent this is to look for haplotypes in which allelic variants are either uniquely embedded or uniquely linked to a trait. However, reconstructing haplotypes from separate SNP assays for genic regions of interest is not straightforward in polyploids^10^. Obtaining haplotypes directly from sequencing reads is possible, but depends on SNP density, requires sufficient read depth, and is sensitive to sequencing errors^22^. Allele dosage inference from sequencing reads also is not straightforward, although methods and tools to deal with this are being developed^24^.

### Pyramiding *R* genes

Pyramiding of *R* genes in a single plant genotype is important to increase the durability of disease resistance^144,145^. Pyramiding of *R* genes is complicated by the fact that diagnostic pathotypes are required in phenotypic assays for the presence of a particular *R* gene. Interactions between host genotypes and genotypes of the pathogen are often complex, involving multiple factors. Many pathotypes can be recognized by different *R* genes, making them unsuitable as diagnostic pathotypes. As a consequence, DNA markers linked to specific R loci or those which are derived from the gene itself would be required to pyramid *R* genes in roses and other crops. In roses, markers for *Rdr1* have been derived from the gene directly^146^, whereas markers for *Rdr3* and *Rdr4* have been discovered by mapping these genes to particular regions of the rose genome^33,114^. Of particular interest are combinations of *Rdr1* and *Rdr4* as each of these genes confer broad-spectrum resistance to a range of black spot pathotypes. Furthermore, the genes have different genetic backgrounds and lie on different chromosomes. The region around *Rdr4* does not contain sequences similar to *Rdr1* (T.D. and M.L., unpublished results), which makes it less likely that the combined resistance specificity against most known isolates can be broken easily by pathogen adaptation.

Besides the difficulty of phenotypically distinguishing the effect of several resistance alleles, pyramiding resistances could create further problems. By combining different resistances from different breeding material (especially non-elite material, e.g. the introgression of *Rdr4* from a climber rose to cut rose), many undesired alleles at other loci affecting other traits may be introduced. This linkage drag may necessitate multiple rounds of back-crossing and selection to recover resistant elite lines. Luckily, this process can be greatly accelerated using closely linked markers (coupled with a marker set which captures the elite genetic background). Owing to the genome sequence and the ease with which the donor and receptor plants can be re-sequenced, it should now be relatively straightforward to saturate regions surrounding *R* genes with SNP markers, and enable marker-assisted back-crossing and gene pyramiding. In addition, GWAS using the information of ultra-dense SNP maps combined with the available genome sequences will enable the detection of minor resistance factors, which can be added to resistance pyramids.

### QTL analysis, association analysis and genomic selection

QTL analysis studies often had a relatively low genetic resolution due to the limited population sizes used. In addition, only alleles present and segregating between the parents of the cross can be detected in such bi-parental studies. GWAS offer an alternative approach to identify genomic regions associated with specific traits in more diverse populations with potentially wider pools of interesting alleles. However, GWAS in rose panels that include multiple types of roses, or multiple species may suffer from serious confounding between population structure and allelic effects, leading to a loss of discriminatory power. Populations connected through common parents offer a compromise by enriching allelic diversity within the context of a balanced and controlled population structure^56^. They also reflect the types of populations generated during a breeding programme. Combining breeding and research in this way would represent an efficient use of resources.

Traits such as vase life, which cannot be scored in young plants, probably represent the first set of candidates for the deployment of molecular tools within a breeding programme. However, such traits are often complex or quantitative, for which molecular tools are less easily developed. A first step could be an analysis of the physiological components of vase life ending, as was done in chrysanthemum^147^. Depending on this, pertinent variables of vase life that may be more accurately phenotyped may be found. Subsequently, these determinants may be studied at the level of their underlying gene pathways. Given the complexity of such traits, genomic selection may prove to be a more appropriate approach. Genomic selection is an alternative method of increasing genetic gain in plant breeding programmes^148^. Whether it is suited to rose has yet to be demonstrated—as rose breeding involves the mixing of diverse genetic backgrounds that may distort the modelling of relationships in prediction models.

### Tailored breeding of rose

Breeding goals in roses depend on their usage. In cut roses, production is a primary breeding goal and has yet to reach its full potential. Breeding companies differentiate their selection to specific production areas, resulting in the best-adapted cultivars for specific environments^149^. Disease resistance is needed to reduce the use of pesticides. Because of post-harvest transport of roses from areas of cultivation (e.g. in Africa and or South America) to consumers all over the world, diseases are not limited to the production environment, but also include post-harvest diseases such as grey mould (*Botrytis cinerea*). Likewise, stress tolerance is important during cultivation, but also for vase life. Rose breeding therefore needs to incorporate many traits, ideally ones that are stably expressed across environments^34,120^.

Fragrance is gaining new interest because of consumer demand and the improved understanding of its underlying genetics. New traits in cut roses include novel flower shapes, colour evolution and glossiness of the foliage, and so on. In pot roses, plant habitus, flower colour, size and number of flowers per stem, number of petals, production and shelf life are the most important traits^149^. In garden rose, disease resistance, fragrance, abundant, red and everblooming flowers were the traits of most interest among surveyed consumers^150^. Carefree roses are needed for landscaping and urban development - these are own-rooted roses supporting mechanical pruning, free of diseases and having an all-season decorative effect, for example, with colourful hips during wintertime. In the near future, because of climate change and expanding trade to more ‘difficult’ areas we may also need cultivars that have improved abiotic stress resistance, including frost tolerance and flowering during hot summers.

Clonally propagated plants can be easily replicated to establish a multi-environment set-up. Larger mapping populations, preferably replicated across multiple different environments, allow for genotype × environment interaction to be studied and understood. This may include RNA-sequencing eQTL mapping in GWAS^151^.

Gene editing with CRISPR/Cas is interesting for rose as a novel tool for directed mutagenesis, as it can produce homozygous mutations in a polyploid^152–154^. Apart from restricted access by IP rights and constraints raised by legal issues in the EU^155^, the application of such new breeding techniques critically depends on the knowledge about gene functions. Although reference genome sequences are currently available, an approach using phenotypic and ‘omics’ data is needed in crops as the vast majority of genes remains uncharacterized^156^. Especially when a loss of function is aimed for, specific information on the targeted sequences is needed.

## Conclusions

In the Rosaceous crops apple and peach, an integrated approach was carried out to fill the gap between available genome data and breeding in an EU-funded project: FruitBreedomics. Complementary approaches were used, including the development of tools and software, to help unravel the genetic control of the most important horticultural traits, as well as to develop plant material and methodologies for breeders. The project started through a consultation between breeders and researchers, aimed at cataloguing the most important traits. Overall, the project led to the development of new phenotyping tools to assess traits like fruit texture, and both biotic and abiotic stresses. Pre-breeding material with specific characterized traits was made available for breeders together with tools for the implementation of molecular markers in breeding^157^. Such a project may well help bridge the gap that still exists between rose research and breeding.

The slow adaptation of molecular techniques can, in some crops, be linked to the long breeding cycle (e.g. in fruit trees^157^), although poplar is an example of a tree with a long breeding cycle in which genomic studies were initiated many years ago^158^. For roses, breeding cycles are not particularly long, but a common platform for interaction between the different players (research and breeders) has been lacking. The recent rose genome sequencing projects that have been conducted were largely driven by research groups without support from the breeding industry. On the other hand, the WagRhSNP array, the high-density genetic maps, and GWAS studies have been developed with support of rose breeding companies.

The new tools (e.g. genome sequence, SNP array, software for dosage scoring and genetic mapping, etc.) and technology (e.g. next-generation sequencing) means that a new era has started for research in polyploid crops such as rose. Previous results on QTLs can be tied together, and new studies can build on this resource. Most resources are freely available at the GDR website^13^.

Genomic studies have opened up new routes to crop improvement, and this holds also for rose. We expect that researchers will use the resources to achieve faster progress in the fields described above. We hope that rose breeders will team up with researchers to discuss their needs and to start to incorporate marker-based selection methods to improve rose breeding for new demands within their breeding programmes.

## Supplementary information

Supplementary Information
