# Supplementary material for: In the name of the rose: a roadmap for rose research in the genome era
Source: Hortic Res. 2019 May 3;6:65. doi: 10.1038/s41438-019-0156-0 (PMC6499834; doi:10.1038/s41438-019-0156-0)
Supplement: Supplementary file 1 — Supplementary Information [file 41438_2019_156_MOESM1_ESM.docx]

**Suppplementary Table 1.** Overview of efficiency, target and process used in stable Rose genetic transformation

| Reference | Cultivar | Ploidy | Modified character | Transformation by | Integrated gene | Target tissus | Efficiency of transformation |
| --- | --- | --- | --- | --- | --- | --- | --- |
| Firoozabady *et al.* 1994 | ‘Royalty’ | 4X | Flower color | *A. tumefaciens* | *Chalcone synthase* | Embryogenic callus | ND |
| Matthews *et al.* 1994 | *Rosa persica* X *xanthina* | 4X | Marker Gene | *A. tumefaciens* | *β-glucuronidase (intron)* | Protoplastes | ND |
| Derks *et al.* 1995 | ‘Sonia’ | 4X | Disease resistance | *A. tumefaciens* | *β-glucuronidase (intron), cecropin B* | Embryogenic callus | ND |
| Souq *et al.* 1996 | *‘Deladel’* | 4X | Plant Architecture  and Flower color | *A. tumefaciens* | *Chalcone synthase* | Embryogenic callus | 1-2 % |
| Van der Salm *et al.* 1997, 1998 | ‘Moneyway’ | 4X | Root system | *A. rhizogenes* | *Rol gene* | Embryogenic callus | ND |
| Marchant *et al.* 1998 | *‘Glad tidings’* | 4X | Disease resistance | biolistic | *β-glucuronidase (intron)* | Embryogenic callus | ND |
| Dohm *et al.* 2001, 2002 | ‘Heckenzauber’ et ‘Pariser charme’ | 4X | Disease resistance | *A. tumefaciens* | *Chitinase Glucanase Lysozyme* | Somatic Embryo | ND |
| Li *et al.* 2002 | *‘Carefree beauty’* | 4X | Marker Gene | *A. tumefaciens* | *β-glucuronidase* | Somatic Embryo | ND |
| Condliffe *et al.* 2003 | ‘Only love’ ‘Romy’ ‘Fresco’ ‘Tineke’ ‘Glad Tidings’ | 4X | Marker Gene | *A. tumefaciens* | *β-glucuronidase (intron)* | Somatic Embryo | ND |
| Li *et al.* 2003 | *‘Carefree Beauty’* | 4X | Disease resistance | *A. tumefaciens* | *Ace-AMP1* | Embryogenic callus | 0,09 |
| Kim *et al.* 2004 | ‘Tineke’ | 4X | Marker Gene | *A. tumefaciens* | *GFP* | Embryogenic callus | 6,6% |
| Chen *et al.* 2006 | *Rosa chinensis* Jacq. | ? | Marker Gene | *A. tumefaciens* | *β-glucuronidase (intron)* | organogenic callus | ND |
| Vergne *et al.*, 2010 | *R chinensis* ‘Old Blush’ | 2X | Marker Gene | *A. tumefaciens* | *β-glucuronidase (intron)* | Embryogenic callus | 3-9% |
| Zvi *et al.*, 2012 | *‘Pariser charme’* | 4X | Transcription factor | *A. tumefaciens* | *PAP1* | Embryogenic callus | ND |
| Zakizadeh *et al.*, 2013 | Rosa hybrida ‘Linda’ | 4X | Marker Gene | *A. tumefaciens* | *P_SAG12_-ipt* | Embryogenic callus | 0,1 |
| Xing *et al.*, 2014 a, b | *R. rugosa* | 2x | Flowering | A. tumefaciens | *β-glucuronidase FT Prunus mume* | Embryogenic callus | 0,00114 |
| Randoux et al, 2014 | Rosa hybrida ‘RI’ | 2x | Flowering | *A. tumefaciens* | *RoKSN* | Embryogenic callus | 0.17-12% |
| Qui et al, 2015 | *Rosa multiflora* | 2X | Disease resistance | *A. tumefaciens* | *MLO genes* | Embryogenic callus | ND |
